# An Efficient and Effective Framework for Intestinal Parasite Egg Detection Using YOLOv5

**DOI:** 10.3390/diagnostics13182978

**Published:** 2023-09-18

**Authors:** Satish Kumar, Tasleem Arif, Gulfam Ahamad, Anis Ahmad Chaudhary, Salahuddin Khan, Mohamed A. M. Ali

**Affiliations:** 1 Department of Information Technology, BGSB University, Rajouri 185131, India; 2 Department of Computer Sciences, Baba Ghulam Shah Badshah University, Rajouri 185131, India; 3 Department of Biology, College of Science, Imam Mohammad Ibn Saud Islamic University (IMSIU), Riyadh 11623, Saudi Arabia; 4 Department of Biochemistry, College of Medicine, Imam Mohammad Ibn Saud Islamic University (IMSIU), Riyadh 11623, Saudi Arabia; 5 Department of Biochemistry, Faculty of Science, Ain Shams University, Cairo 11566, Egypt

**Keywords:** intestinal parasites, transfer learning, CNN, YOLOv5

## Abstract

Intestinal parasitic infections pose a grave threat to human health, particularly in tropical and subtropical regions. The traditional manual microscopy system of intestinal parasite detection remains the gold standard procedure for diagnosing parasite cysts or eggs. This approach is costly, time-consuming (30 min per sample), highly tedious, and requires a specialist. However, computer vision, based on deep learning, has made great strides in recent years. Despite the significant advances in deep convolutional neural network-based architectures, little research has been conducted to explore these techniques’ potential in parasitology, specifically for intestinal parasites. This research presents a novel proposal for state-of-the-art transfer learning architecture for the detection and classification of intestinal parasite eggs from images. The ultimate goal is to ensure prompt treatment for patients while also alleviating the burden on experts. Our approach comprised two main stages: image pre-processing and augmentation in the first stage, and YOLOv5 algorithms for detection and classification in the second stage, followed by performance comparison based on different parameters. Remarkably, our algorithms achieved a mean average precision of approximately 97% and a detection time of only 8.5 ms per sample for a dataset of 5393 intestinal parasite images. This innovative approach holds tremendous potential to form a solid theoretical basis for real-time detection and classification in routine clinical examinations, addressing the increasing demand and accelerating the diagnostic process. Our research contributes to the development of cutting-edge technologies for the efficient and accurate detection of intestinal parasite eggs, advancing the field of medical imaging and diagnosis.

## 1. Introduction

Intestinal parasitic infections have significant implications for public health worldwide, particularly in developing and underdeveloped countries. According to the World Health Organization, infectious and parasitic diseases affect approximately 24% of the global population, and in 2020, preventive chemotherapy was administered to 836 million children globally [1]. These infections often result in diarrhea, malnutrition, anemia, and other symptoms, primarily affecting children. Over 100 species of intestinal parasites reproduce daily, hatching at a rate of 200,000 eggs per day. Manual light microscopy serves as the standard method for diagnosing parasitic diseases [2,3]. However, parasite species identification and quantification based on microscopy are complex, labor-intensive, and time-consuming processes that require both a microscope and expert knowledge [4,5]. Additionally, analyzing microscopic images poses challenges for human experts due to the variations and uncertainties in morphological features, such as shape, staining color, and density of parasite species.

Previously, the automation of analyzing intestinal protozoa was limited due to the lack of automatic recognition algorithms for microorganisms under the microscope. However, in recent years, deep learning architectures have revolutionized various machine-learning tasks, particularly in the field of medical image identification and classification. Among these architectures, convolutional neural networks (CNNs) have played a crucial role in significantly improving performance [6,7,8]. Unlike traditional machine learning methods, deep learning architectures directly extract features from images, leading to enhanced validation and detection accuracy [9,10]. Deep learning applied to the detection of plasmodium (malarial parasite) in blood has achieved detection performance close to that of human experts [11,12,13]. Amal H. A. et al. developed a deep learning-based model by conducting experiments on 13,750 parasitized and 13,750 non-parasitic samples, achieving an estimated accuracy rate of 97% for recognizing the samples [14]. In a recent study by Z. Jing et al. [15], a novel approach for fecal cell detection was presented using the Faster R-CNN model combined with the powerful Resnet-152 convolutional neural network architecture. The proposed algorithm demonstrated impressive accuracy, achieving an average precision of 84% on a dataset of 40,560 fecal images. Additionally, the detection time per sample was significantly reduced to only 723 ms, enabling rapid analysis and diagnosis of fecal specimens in clinical settings.

**Figure 1:**
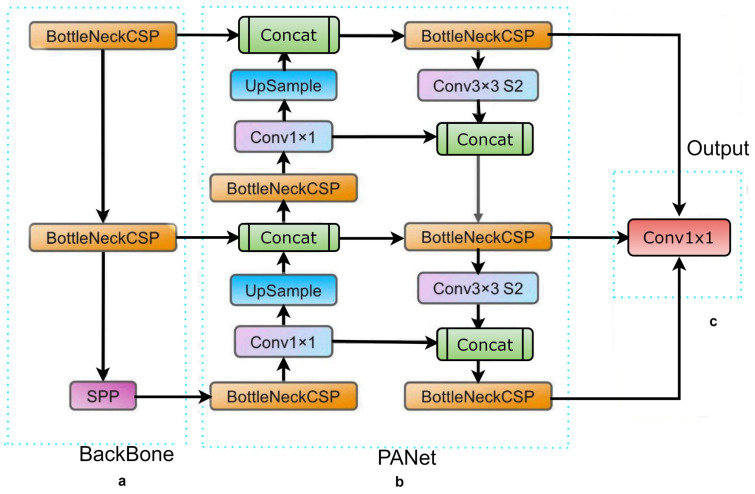
The YOLOv5 network architecture comprises three essential components: (**a**) the BackBone (CSPDarknet), (**b**) the Neck (PANet), and (**c**) the Output (YOLO Layer). The process begins by feeding the data into the Backbone, which performs feature extraction. Subsequently, the extracted features are passed to the PANet for feature fusion. Finally, the YOLO Layer generates detection results based on the fused features.

Here, we address the issue of image pre-processing and augmentation for rapid automatic detection and classification in raw parasite images. We have adopted a multi-step routine for data preparation, neural network configuration, model training, and prediction analysis. We applied the You Only Look Once (YOLO) CNN-based model for object detection [15,16,17], specifically the YOLOv5 architecture for detection and classification of samples in the dataset. YOLO is a cutting-edge, real-time object detector, and YOLOv5 builds on YOLOv1-YOLOv4. It has consistently outperformed on two official object detection datasets: Pascal VOC (visual object classes) [18] and Microsoft COCO (common objects in context) [19]. Figure 1 depicts the YOLOv5 network architecture. We have utilized YOLOv5 in this experiment as our initial learner for three reasons. First, YOLOv5 came with CSPDarknet, the backbone of Darknet, including a cross stage partial network (CSPNet) [20] into it. CSPNet minimizes the model’s parameters and FLOPS (floating-point operations per second), while ensuring inference speed and accuracy, while also reducing the model’s size by integrating gradient changes into the feature map and resolving issues with repeated gradient information in large-scale backbones. The task of detecting intestinal parasites required speed and accuracy for detection, and the efficiency of inference on edge devices with limited resources is also derived by the compact model size. The YOLOv5 has used the path aggregation network (PANet) [21] to improve information flow in its neck. PANet utilised a novel feature pyramid network (FPN) structure with an improved bottom-up path that greatly enhances the transmission of low-level features. Furthermore, adaptive feature pooling is used to link feature grids at all levels and permit essential information from each feature level to extend to the next subnetwork. The PANet approach improves the utilization of precise localization signals in down layers, which increases the object’s location accuracy. Third, the YOLO layer, which serves as the powerhouse of YOLOv5, generates feature maps in three different sizes (18 × 18, 36 × 36, and 72 × 72), giving the model the ability to handle objects of varying sizes, from small to medium to large. In a similar way, the growth of a forest fire typically progresses from a small-scale ground fire to a medium-sized trunk fire, and eventually to a large-scale canopy fire. By utilizing multi-scale detection, the YOLOv5 model is able to accurately track these size changes, allowing for faster and more effective fire suppression efforts.

We utilized data augmentation techniques on the source parasite images to generate more regularization and divergence in the training dataset. We attempt to optimize the model by applying an additional dataset and using augmentation and transformation to enhance the results. We were able to accurately detect parasites from a separate test set of microscopic images by using trained YOLOv5 algorithms.

## 2. Related Work

Previous efforts in the computational diagnosis of intestinal parasitic infections have primarily focused on improving detection accuracy through hand-engineered feature extraction techniques, which require specialized skills and experts [22]. However, recent developments in pre-trained deep learning models have the potential to revolutionize medical image analysis, especially in scenarios where data are limited and heterogeneous [23]. These models [24,25,26] can serve as feature extractors for a new model trained on a dataset with fewer images, improving performance in different tasks of computer vision. Recent studies have applied pre-trained deep learning models to the classification and detection tasks of differentiating infected parasitized from microscopic images, achieving classification and detection accuracies above 95% and outperforming traditional machine learning algorithms [27]. However, most of these studies have focused on the detection tasks of intestinal parasite images, which are less sensitive and may miss cysts or eggs of parasites due to morphological features. Therefore, there is a need to scale up the approach of transfer learning for object detection in intestinal parasitic infections, which could provide more insights and considerably improve parasite detection.

In recent years, the You Only Look Once (YOLO) model for object detection has gained significant attention and has been continuously developed. YOLO directly detects multiple objects by predicting multiple bounding boxes and class probabilities, making it a popular one-stage detection model [15,17,28]. With subsequent improvements in accuracy and computational performance, YOLO-based architectures, such as YOLOv4 [29], YOLOv5 [30], and YOLOv8 [31], have been successfully applied in various research works. Although YOLOv8 is the latest version, there is still ongoing debate about the comparisons between the YOLO versions. While YOLOv5 has higher precision and speed than other state-of-the-art algorithms, such as Faster R-CNN [32] and SSD [26], YOLOv5 has higher accuracy and detection speed compared to YOLOv4 and YOLOv8. However, due to the complexity of the YOLOv8 network, a more simplified version of YOLOv5 has been designed to maximize detection speed and improve computational efficiency. This model has been applied in various applications, including the detection of pine wilt disease, trash, and electronic components. In our research, we focus on the YOLOv5 because of its faster detection results and memory usage with low-end GPU devices. We propose using this model for the automatic recognition of six common classes of protozoan cysts and helminthic eggs in parasitic products of stool examination, which is suitable for remote areas with limited laboratory testing capabilities. By utilizing the YOLOv5 model, we aim to improve the detection and classification of protozoan cysts and helminthic eggs for more effective and prompt treatment.

## 3. Material and Methods

### 3.1. Dataset Collection

**Figure 2:**
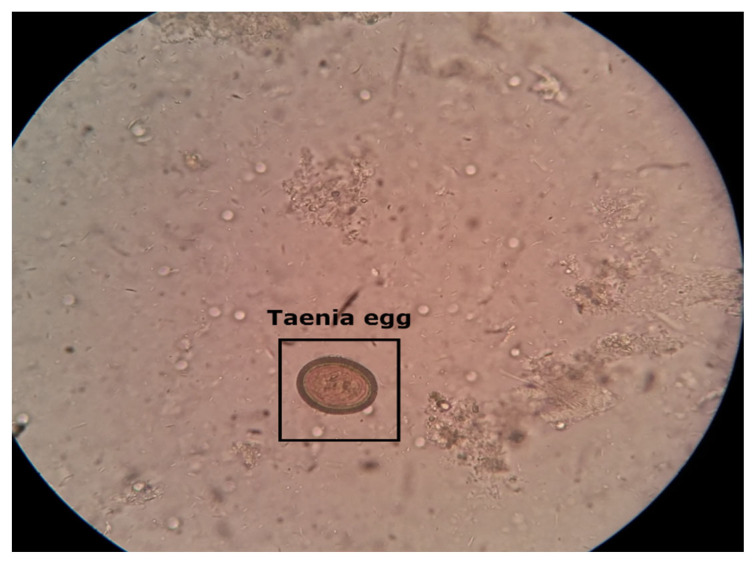
Microscopic stool sample of Taenia parasite image captured with a smartphone. Taenia intestinal parasite belonging to the genus Taenia that primarily infects the intestines of humans and other animals.

The intestinal parasite dataset used in this research report contains 10× magnification microscopic images with a resolution of 416 × 416 pixels. Five different types of intestinal parasites cysts or eggs collect *hookworm eggs*, *Hymenolepsis nana*, *Taenia*, *Ascaris lumbricoides*, and *Fasciolopsis buski*. These images were obtained from Mulago Referral Hospital, located in Uganda, as well as the IEEE Dataport [33,34]. The intestinal parasite images were annotated using the open-source data annotation graphical user interface (GUI) tool “Roboflow” (https://app.roboflow.com/) (accessed on 17 April 2023). Figure 2 depicts microscopic stool images captured using smartphones. The test performance of the model can be affected by the small number of images available for training. Therefore, to reduce the effect of overfitting after model training, data augmentation techniques were used, which include vertical and rotational augmentation.

The images were resized to 416 × 416 for the YOLOv5 algorithms. Finally, the dataset split into training, validation, and testing in the ratios of 70%, 20%, and 10%, respectively.

### 3.2. Data Preparation

**Figure 3:**
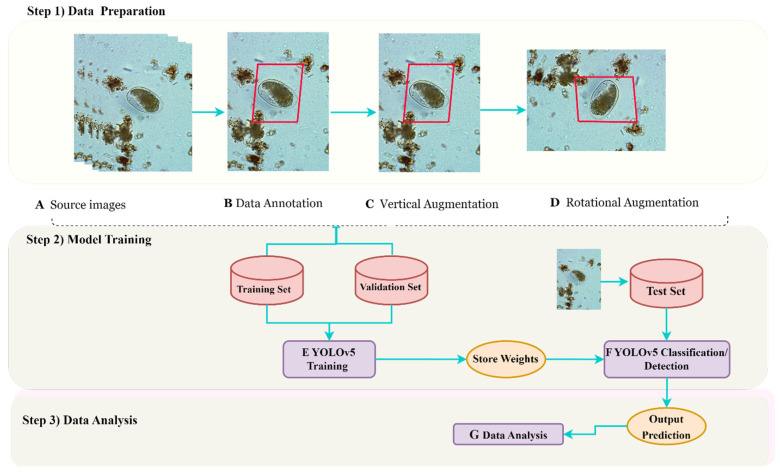
This flow chart illustrates the process of data preparation and analysis for utilizing YOLOv5s and YOLOv5l models. Step 1) initiates with the collection of parasite images, which are subsequently annotated and subjected to data augmentation techniques, such as vertical and rotational methods. Moving on to Step 2), the YOLOv5 models are trained using both training and validation datasets, after which PyTorch weight files are applied to optimize their performance in predicting on the test dataset. Utilizing the trained weights, the YOLOv5 detection and classification function is employed to generate output predictions. Finally, in Step 3), advanced data analysis techniques are employed to evaluate the models’ performance. Our innovative flow chart showcases the remarkable possibilities that arise from the combination of advanced technology and data analysis, leading to a revolution in parasite detection and significant enhancements in global healthcare.

Figure 3 provides a comprehensive overview of the innovative approach implemented for parasite image detection, encompassing essential stages, such as data preparation, annotation, augmentation, training, evaluation, and data analysis. To annotate the parasite images, we utilized the advanced data annotation tool called “Roboflow”. This tool offered a user-friendly graphical user interface (GUI) to accurately draw bounding boxes around both positive and negative images of intestinal parasites. Each bounding box encompassed the entire parasite species depicted in the image, and the annotations were saved in YOLO format, accompanied by corresponding text files. To create a more diverse and robust training dataset, we employed a widely used technique called data augmentation. This involved applying various transformations to each source image, resulting in a larger and more varied dataset for training. The resulting dataset comprised a substantial number of unique images, which served as the training dataset. For evaluation purposes, we utilized a separate test dataset, consisting solely of unannotated parasite images to assess the performance of our model. With this innovative approach, we have confidence that our method will contribute to accurate and efficient parasite detection, bringing us closer to the eradication of parasitic infections. Section 3.3 focuses on deep-learning, specifically YOLOv5 training and prediction.

### 3.3. Deep Learning: YOLOv5 Training and Prediction

In a recent study, we employed advanced machine learning techniques, specifically YOLOv5 algorithms, to achieve unparalleled accuracy and efficiency in detecting parasites. These state-of-the-art algorithms allowed us to extract the optimal weights from our extensive dataset of 5393 parasite images, encompassing various resolutions. To enhance our results, we conducted experiments with different variants of the YOLOv5 algorithm and trained our models using both YOLOv5s and YOLOv5l object identification models.

We selected these particular models based on their ease of implementation on freely available cloud computing platforms, such as the Google Collaboratory, making them accessible to a wider audience. Additionally, the YOLOv5 models offer a diverse range of pre-written model architectures, each tailored to specific speed and accuracy requirements under different conditions. Our models were trained using a dataset of approximately 950 MB, and the training process took only one hour on a Tesla T4 GPU(China). The learned weights and biases of our models were automatically saved into a PyTorch weights file, simplifying the process of reusing and improving our results.

As shown in Figure 3, this study showcases the potential of these advanced techniques to revolutionize parasite detection, leading to improved healthcare outcomes worldwide and potentially saving numerous lives. Accurate and efficient parasite detection is particularly crucial in regions with limited access to healthcare resources, as it can have a significant impact on disease prevention and treatment efforts.

The YOLOv5 object recognition version was cloned from the Ultralytics YOLOv5 catalogue [35]. The YOLOv5s and YOLOv5l models were trained using the train.py function for 100 epochs with an image size of 416 × 416 and batch size 16. The models were used for both the classification and detection tasks. Both methods were trained with only the default hyperparameters. On the test dataset, these trained weights were then used to make predictions. A 416 × 416 image was used for testing using the detect.py function. Utilizing hardware from the Google Collaboratory cloud computing service, models were trained and tested.

In conclusion, this groundbreaking study highlights the power of advanced machine learning techniques in addressing real-world challenges. By utilizing state-of-the-art algorithms and leveraging cloud computing platforms, we can enhance our ability to detect and combat diseases more effectively. This study is a promising step towards transforming healthcare outcomes globally and advancing the field of machine learning.

### 3.4. Experimental Platform

**Figure 4:**
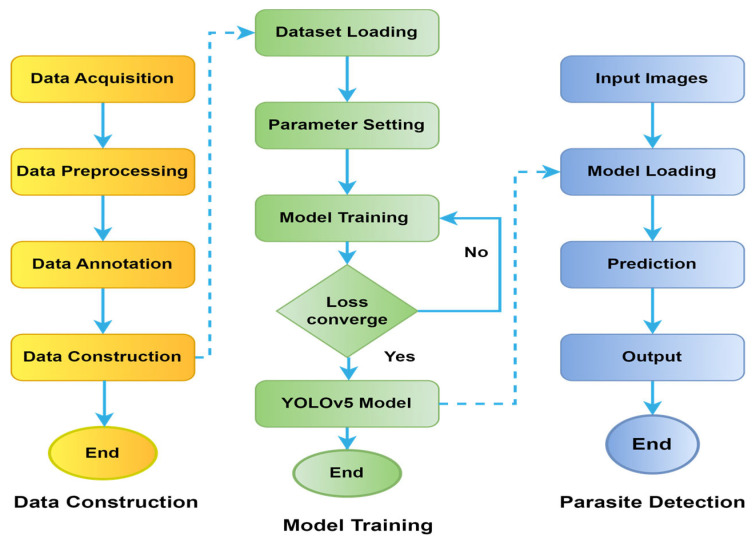
Flowchart of YOLOv5 model for dataset, training, and detection processes.

The experiment was executed on the Google Colab System as the base environment with a Nividia K80/T4 GPU (Taiwan) at 0.82 GHz/1.59 GHz GPU memory clock and GPU memory of 12 GB/16 GB. This experiment was carried out with models built on PyTorch [36], which provides libraries for building the main architecture of a deep learning model. The training of the models was performed using the open-source library Pytorch. Figure 4 represents a detailed flowchart of the dataset, as well as training and detection processes of the YOLOv5 model used in this experiment.

### 3.5. Statistical Analyses

To determine the strength of the conducted experiments on the trained YOLOv5 algorithms, Precision, Recall, F1-score, and mAP measures are used for evaluation. The computation methods are provided in Equations (1)–(5).

(1) $$\mathrm{Accuracy}=\frac{\mathrm{TP}+\mathrm{TN}}{\mathrm{TP}+\mathrm{TN}+\mathrm{FP}+\mathrm{FN}\;}\;$$

(2) $$Recall=\frac{TP}{TP+FN\;}\;$$

(3) $$Precision=\frac{TN}{FP+TN\;}\;$$

(4) $$F1-Score=2\;*\;\frac{Precion\;*\;Recall}{Precion+Recall}\;$$

In this context, the abbreviations *TP*, *TN*, *FN*, and *FP* represent True Positive (correct detections), True Negative, False Positive (incorrect detections), and False Negative (missed detections), respectively. The *F*1-score metric, defined in Equation (3), provides a comprehensive evaluation of the trained model by considering the trade-off between Recall and Precision. In addition to the *F*1-score, the Average Precision (AP) Equation (4) is employed to showcase the overall performance of the models across different thresholds. The following is shown below:

(5) $$AP=\frac{1}{n}\sum_{k=1}^{k=n}P(r)\Delta r\;$$

where; P(r) represents the precision at a given recall level *r,* and Δr denotes the change in recall from the previous level. This formulation aligns with established practices and provides a more meaningful representation of the Average Precision metric.

## 4. Experiment Results

In our pursuit to advance parasite detection, we have gathered a diverse dataset of 5883 images featuring various parasites, including *hookworm eggs*, *Hymenolepis nana*, *Taenia*, *Ascaris lumbricoides*, and *Fasciolopsis.* Our collection spans across continents, with images acquired from the Mulago Referral Hospital in Uganda and the IEEE Dataport.

With this dataset, we developed a novel detection algorithm using YOLOv5, with each image in the development dataset meticulously annotated with vibrant rectangular boxes as the ground truth. To further improve the robustness of our model, we applied data aumentation and split our dataset into training and testing sets. As we trained our models, we experimented with various image resolutions and settled on 416 × 416 pixels to achieve optimal performance.

The YOLOv5 algorithms demonstrated improved detection results for intestinal parasites in the test dataset collection. We computed and compared the precision, recall, F1-score, and AP (average precision) of the detected parasites between the YOLOv5s and YOLOv5l models. The training process utilized the SGD optimizer and lasted for 100 epochs, being completed in 1.04 h for YOLOv5l and 0.918 h for YOLOv5s. Additionally, we employed YOLOv5 features to augment the training images by cropping, adjusting the dynamic range, and changing the scale during the training process. YOLOv5 applied image space and color space augmentations within the training dataset, presenting an original image plus three random images each time an image was loaded for training.

**Figure 5:**
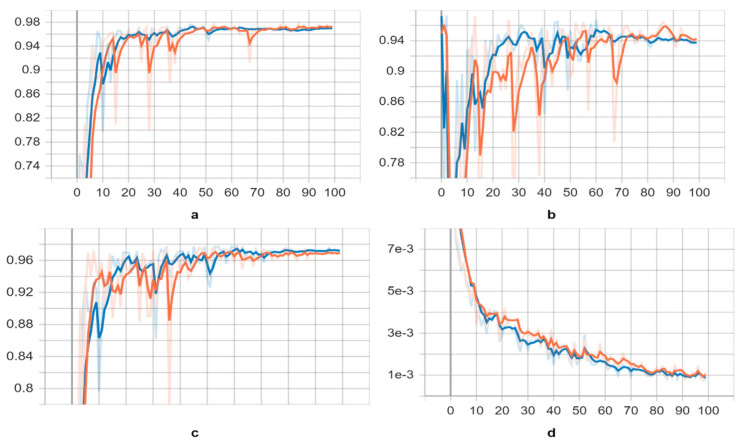
Learning curves of the YOLOv5s and YOLOv5l trained models on parasite dataset, yellow and blue, respectively. As a metric, we used the (**a**) mean Average Precision (mAP) (0.5) score, (**b**) precision score, (**c**) recall score, and (**d**) class loss score.

Initially, we focused on training the “s” and “l” variants of the YOLOv5 model using the training datasets. We selected the weights with the best available scores as the key indicators of the overall training stage. The scores of the trained neural networks exhibited a significant increase in the 20th and 24th epochs for both models, while the corresponding loss decreased (refer to Figure 5). These epochs marked the point at which the neural networks began identifying parasite species. However, as the training processes of both models extended to 100 epochs, the validation scores gradually declined after the 90th epoch, and the trained deep neural networks were no longer able to recognize parasites effectively.

**Figure 6:**
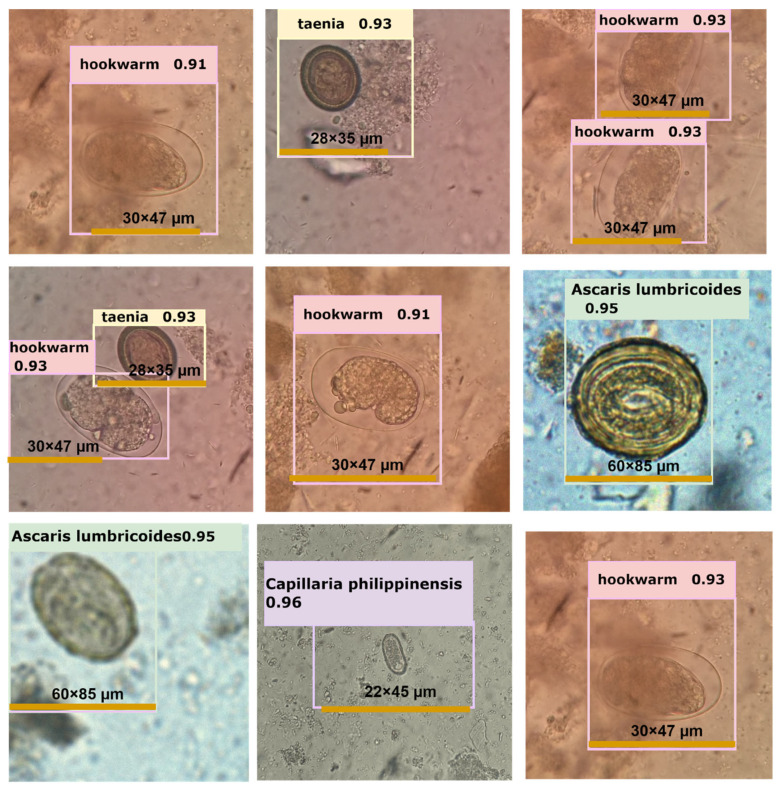
Depiction of several examples of intestinal parasite detection performed by YOLOv5l neural networks.

After applying pre-trained weights to both YOLOv5 models for validation, we obtained results presented in Table 1 and visualized in Figure 5. The trained neural networks exhibited improved recognition of parasite species, with slightly higher average mAP (0.5), mAP (0.5:0.95), recall, and precision scores, as indicated in Table 2. In Figure 6, we provide examples of parasite species detection by YOLOv5l models, showcasing their impressive performance, while also highlighting significant distortion in the bounding boxes around the detected species. It is worth noting that the evaluated performance varies depending on the approach used. Although the YOLOv5l model demonstrates higher accuracy, it also possesses more layers and parameters, resulting in slower processing compared to the YOLOv5s model, as illustrated in Table 3. Overall, our findings suggest that the YOLOv5 models are effective in detecting parasite species, offering valuable insights for future research in this field.

### Comparison with Object Detection Models

We evaluated the performance of our proposed methods against four state-of-the-art object detection models: single-shot detector (SSD) [18], Faster R-CNN [19], AlexNet, and ResNet50. While SSD is a lightweight network that detects multiple objects in a single shot, Faster R-CNN requires two shots for object detection—one to identify regions of interest (ROI) and another to detect the object in each ROI using CNNs. We used VGG-16 and ResNet50 as the backbones for SSD and Faster R-CNN, respectively.

Table 4 summarizes the precision results of our methods compared to these four models. Interestingly, our proposed YOLOv5 model outperforms Faster R-CNN with the best average precision. Our YOLOv5 model also outperforms SSDs, despite having a smaller architecture and fewer parameters. The sliding window technique employed in our method works well for parasite egg detection, as it allows us to fix the size of each patch. Unlike natural images containing objects with various shapes and aspect ratios, parasite eggs have a consistent aspect ratio. Thus, the adaptive size of a bounding box offered by SSD and Faster R-CNN does not improve parasite egg detection.

## 5. Discussion

This research paper delves into the utilization of advanced deep learning techniques and cloud computing platforms to enhance parasite detection. The study employed a diverse dataset of 5883 images, encompassing various parasites, such as *hookworm eggs*, *Hymenolepis nana*, *Taenia*, *Ascaris lumbricoides*, and *Fasciolopsis.* These images were sourced from the Mulago Referral Hospital in Uganda and the IEEE Dataport. The researchers developed a novel detection algorithm using YOLOv5 and meticulously annotated each image in the development dataset with vibrant rectangular boxes as ground truth. The dataset was split into training and testing sets, and data augmentation techniques were applied to enhance the model’s robustness. The YOLOv5 algorithms exhibited favorable detection results, with Precision, Recall, F1-score, and AP metrics calculated and compared between the YOLOv5s and YOLOv5l models. Image and color space augmentations were implemented in the training dataset, presenting an original image plus three random images each time an image was loaded for training.

The study trained both the “s” and “l” variants of the YOLOv5 model using the training datasets, and the best weights were selected based on the overall training stage. The scores of the trained neural networks experienced a significant increase in the 20th and 24th epochs for both models, accompanied by a corresponding decrease in loss, indicating that the neural networks started to identify parasite species. However, as the training process extended to 100 epochs, the validation scores gradually declined after the 90th epoch, and the trained neural networks were unable to effectively recognize parasites.

Upon applying pre-trained weights to both YOLOv5 models for validation, the trained neural networks exhibited improved recognition of parasite species, demonstrating slightly higher average mAP (0.5), mAP (0.5:0.95), recall, and precision scores. The YOLOv5l model showcased higher accuracy, but it was slower than the YOLOv5s model due to its increased number of layers and parameters. The researchers provided examples of parasite species detection by YOLOv5l models, showcasing their impressive performance while acknowledging significant distortion in the bounding boxes surrounding the detected species. Overall, the findings of the study suggest that the YOLOv5 models are effective in detecting parasite species and offer valuable insights for future research in this field. The research emphasizes the power of advanced machine learning techniques in addressing real-world challenges and improving disease detection and combat.

## 6. Conclusions

In this study, our main objective was to enhance the performance of two cutting-edge deep-learning architectures, namely, YOLOv5s and YOLOv5l, in the domain of object detection, specifically focused on intestinal parasites. To achieve this, we conducted a comprehensive evaluation of various metrics, including precision, recall, F1-score, and mean average precision. Our assessment was performed on a dataset consisting of 5393 microscopic images of parasites, each with an input image resolution of 416 × 416. Through systematic analysis of these key parameters, our aim was to optimize the algorithms’ capabilities and improve their accuracy in detecting objects. Our findings revealed that YOLOv5l outperformed YOLOv5s in terms of overall accuracy. However, both algorithms demonstrated excellent performance in recognizing and accurately locating the five types of parasites within the images. Notably, the YOLOv5 algorithms exhibited significantly faster processing times compared to Faser-RCNN [37] and ResNet50 [27], which require selective search and consequently consume more time. Interestingly, the mAP performance for the *Hookworm*, *Taenia*, and *Fasciolopsis buski* parasites was outstanding, achieving a score of 0.99. We attribute this success to the distinct characteristics of *hookworms* and the abundance of hookworm images in our dataset, which facilitated effective data enhancement during training. The mAP values for *Ascaris lumbricoides* were also commendable, scoring 0.92 for the YOLOv5 algorithms. However, due to the small dataset size and class imbalance, the training model experienced some degree of overfitting. Despite this limitation, our proposed deep learning model for intestinal parasite detection achieved the highest mAP and demonstrated rapid detection and localization of *hookworms*, *Taenia*, *Fasciolopsis buski*, *Ascaris lumbricoides*, and *Hymenolepis nana,* with a mAP of approximately 97% and a detection time of 8.5 ms per image. Future work should aim to expand the dataset size and include other intestinal parasites in the analysis to address the overfitting issue associated with small dataset sizes. Additionally, our study contributes to the development of effective deep learning models that can be utilized for identifying intestinal parasitic cysts or eggs in stool examinations.

## Figures and Tables

**Table 1:** Overview of the intestinal parasites dataset obtained after applying data augmentation techniques. The dataset is divided into a training set (70%), validation set (20%) and testing set (10%).

Contents	Training Dataset	Validation Dataset	Testing Dataset
# images	3657	1376	850
*hookworm*	827	300	150
*Hymenolepsis nana*	370	130	100
*Taenia*	730	300	26
*Ascaris lumbricoides*	850	320	250
*Fasciolopsis buski*	850	320	250

**Table 2:** Comparison of the YOLOv5s and YOLOv5l neural networks trained, by using transfer learning and pre-trained on trained weights, on validation datasets.

			YOLOv5s				
Classes	Images	Instances	mAP50	mAP50-95	Precision	Recall	Specificity
*Hookworm*	652	180	0.995	0.655	0.959	1	0.921
*Taenia*	652	29	0.995	0.646	0.951	1	0.910
*A. lumbricoides*	652	195	0.933	0.714	0.923	0.909	0.922
*F. buski*	652	198	0.995	0.655	0.959	1	0.980
Total	652	582	0.974	0.685	0.944	0.97	0.992
			**YOLOv5l**				
*Hookworm*	652	180	0.995	0.722	0.952	1	0.935
*Taenia*	652	29	0.993	0.70	0.946	1	1
*A. lumbricoides*	652	195	0.92	0.72	0.915	0.972	0.832
*F. buski*	652	198	0.993	0.70	0.946	1	1
Total	652	201	0.969	0.722	0.938	0.972	0.910

**Table 3:** Parasites’ detection speed in YOLOv5 models.

Model Summary	Layers	Parameters	GFLOPs	Pre-Process Speed	Inferences Speed
YOLOv5s	157	7,020,913	15.8	0.5 ms	8.5 ms
YOLOv5l	267	46,124,433	107.7	0.3 ms	15.4 ms

**Table 4:** Comparison with object detection models.

Models	mAP (%)				
	*Hookworm*	*H. nana*	*Taenia*	*A. lumbricoides*	*F. buski*
SSD	0.857	0.741	0.833	0.873	0.881
AlexNet	0.863	0.800	0.900	0.957	0.830
Faster R-CNN	0.825	0.873	0.989	0.858	0.861
ResNet50	0.862	0.900	0.900	0.900	0.866
Our YOLOv5	0.995	0.995	0.995	0.995	0.995

## Data Availability

The datasets used and/or analyzed during the current experiment for training and testing purposes are available from the corresponding author on reasonable request. All data annotation and augmentation tools are provided by Roboflow at the following link (https://app.roboflow.com/) (accessed on 17 April 2023). The “You Only Look Once” version 5 (YOLOv5) object detection architecture, written in Python by Ultralytics, is freely available under a GPL-3.0 license (https://github.com/ultralytics/YOLOv5) (accessed on 17 April 2023). Two Jupyter notebooks that are interactive implementations of the YOLOv5 methods for training and detection are provided with this publication. All pre-processed data used in this experiment can be made available upon reasonable request. Correspondence and requests for materials should be addressed to K.S.
